# Some of the Uses of the Laboratory in Diagnosis and Treatment of Disease

**Published:** 1913-03

**Authors:** Chas. W. Gould

**Affiliations:** Atlanta, Ga.


### SOME OF THE USES OF THE LABORATORY IN DIAGNOSIS AND TREATMENT OF DISEASE

By Chas. W. Gould, M. D., Atlanta, Ga.

Diagnosis is and always has been one of the most difficult problems in the science of medicine.

Some men acquire a skill and facility in diagnosis, or at least apparent correctness in diagnosis which gains for themselves the reputation of wizards among the laity, and sometimes they even get to believing in themselves to a remarkable degree. One such man one day told me that he believed himself to be possessed of almost occult powers, so accurate was he in his diagnosis. He felt that he could almost see the pathological condition of an individual's organs by merely studying his countenance. It is very doubtful, of course, if he could really recognize a pathological condition of an organ if it was before his eyes at autopsy. The next day, a consultant being demanded by one of his "typhoid" patients, an examination of the urine was suggested, and a cystitis was found, which when properly treated cleared up the "typhoid" in a short time. There are many such men whose greatest qualification consists in the ability to impress their clientele that they are wonders.

But that men are secretly concerned about their ability in accurate diagnosis is shown by the acclaim with which the announcement of a specific and accurate method of diagnosing a disease is accorded.

For instance, the names of Hippocrates and later Avenbrugger are associated with auscultation. Laence with percussion and reverence is granted these names, for removing the diagnosis of diseases of the chest from the wide range of clever guesswork to something approaching precision.

When Louwenhock offered to the world a lens powerful enough to reveal the organisms in the saliva, he opened up a field which Pasteur in chemistry and bacteriology, and Virchow in the realm of tissue pathology entered and worked with avidity. The results were discoveries that have paved the way for the great modern advancement in medicine by the later workers, so that now we have accurate methods of diagnosis in diseases of the kidneys, in tuberculosis, typhoid, syphilis, diphtheria and others. So Koch, Widal, Fraenkel, Wasserman and many others have been placed in the temple of fame for offering us a means of diagnosis for various diseases.

Each discovery has been heralded throughout the medical world with the greatest enthusiasm, showing not only interest, but a certain relief, for herein we had another means of putting our feet on solid ground when this or that disease should be under consideration for our study and diagnosis. So these methods of diagnosis have multiplied as scientists have delved deeper into the wonderful mysteries of the laws of nature. We have the significance of the tuberculin reaction, the various blood pictures, revealed by staining and counting, worked out for us. We have found that certain diseases may be diagnosed by culturing the blood to find the causative organism. The urine, feces and stomach contents may be examined, and the secret workings of the kidneys and digestive organs thereby revealed. Functionating tests of the kidneys put us on a speaking acquaintance with the ability of the kidneys to do their work. Until, in fact, a definite field of laboratory diagnosis has opened up, which is as much a specialty as any of the others, and as impossible for the general man or a specialist in any other branch to master and attend to properly, as it is for a gynecologist to do successful work in eye, ear, nose and throat.

A man in general work has not the time to perfect himself in these various laboratory tests, or if competent, he has not the time to carry them out and do his other work satisfactorily.

Because of this belief, many laboratory men have started laboratories in various parts of the country, and most of them have had a hard fight of it. I may say right here, that personally I have met with a very agreeable surprise in the contrast to the reception which I have received and that which I expected, so this paper is not a personal wail. But then, in some places laboratories are not so fortunate. One man told me that he had worked four years for about an average of $35.00 per month. He had a long hard pull before the physicians of his city realized, as they finally have, the value of that kind of work.

Of course, along with the establishing of the private laboratories, have sprung up public, state and municipal laboratories for the diagnosis of tuberculosis, diphtheria, malaria, typhoid, etc., but their scope is necessarily limited to a few diseases.

What is the cause, then, of the lack of interest of the general professional public in this matter? It cannot be because of the self-confidence of physicians in the diagnostic ability, for the most advanced men in medical knowledge are the best patrons of laboratories. Surely the average practitioner should not feel so cock-sure in his diagnosis, when a man conceded by the medical profession to be one of the most thorough in the country, with the best laboratory facilities and the most capable assistants to aid him in gathering data, a teacher of the art and science of diagnosis, a writer of some of the ablest and most exhaustive books on the subject, admits that he only diagnoses about 35% of the cases as to the cause of death which come to his autopsy.

Is it because of the financial consideration? Some say yes, that people think their doctor ought to know it all and do not wish to pay for outside help. But them people do not object to paying from $10.00 to $100.00 for a consultant to come to their bedside. Is it because the physician does not wish to confess his inability to diagnose or do anything and everything in connection with the case? Surely not, for he hands his obstreperous infant feeding case over to the pediatrician, his bad neurasthemics and T. B. patient go to the sanatoriums, his operable case to the surgeon, and most of the others wend their mournful way to the eye, ear, and stomach specialists, and others better fitted to treat them in that particular ailment, until the general man is fast becoming obsolete. No, it cannot be because he is unwilling to confess his ignorance, because most men nowadays confess their ignorance on everything but a specialty of a specialty of a hundredth part of the body. It cannot be because they dislike seeing the other make something on the case, because they are constantly sending their cases to men who get several times the fee that they themselves would obtain from them.

Is it then, because he doesn't recognize that the laboratory man can be of real assistance to him? It sometimes seems so, because that part of the laboratory work which has been most advertised is becoming more and more patronized—the Wassermann test, for example, urine, and with surgeons, tissue and examinations are often called for. Of course, doctors use the public laboratories for diphtheria, tuberculosis, hook-worm, etc. That is but natural. However, experience in a city laboratory has convinced me that only about 5% of the physicians use the public laboratories, even where they may have the examinations made free.

What is the reason? If one has an amaenia which doesn't respond to treatment, and doesn't know that a white and red blood cell count, with a hemaglobin estimate, and a differential blood count may set him right as to whether that anaemia is primary or secondary, he is not apt to call on a laboratory man to help him out. If a man was graduated before the blood counts were made, he is not to be blamed for not knowing that to count the blood cells one must obtain the fresh blood, drawn directly into a special fluid, or that a differential count is a stained smear, and tells the proportion of the different kinds of blood cells. In a large laboratory, a couple of slides came in one day stuck together by the blood dried between them. The man who sent it in asked for an examination for Malaria Plasmodium, a red and white blood cell count, and a Widal test for Typhoid. He knew of the tests, but not the methods of obtaining the specimens.

How many men know the great value of Blood Cultures in the diagnosis of disease? Early in a case of typhoid, you may obtain a diagnosis which is absolutely certain, much more so than a Widal, and may be made much earlier. But, of course, this has to be made by a laboratory man who must go to the bedside of the patient equipped with a knowledge of the media of food which the typhoid organism loves best. Some valuable work and beautiful results have been obtained lately by treating typhoid with vaccine in the early stages of the disease. As Autogenous vaccines are always better than stock vaccines, it has been suggested that blood cultures would be a good way to get the vaccines. Gonorrhoeal Rheumatism may be diagnosed by blood culture if the proper kind if media is used. The typhoid loves bile, the gonococcus hydrocele or acetic fluid mixed in the diet, blood cultures made into agar, in case of sepsis, reveals in 12 hourse, or over night, whether you have a streptococcic infection or staphylococcic, or whether the pneumococcus is the cause of the septicemia by the effect of the different organisms on the blood agar plater.

While in an army hospital last year, a friend of mine acting as a laboratory man suggested that a blood culture on a case that was giving the skilled men at that place a lot of trouble, and what was their surprise to find the pneumococcus in the blood, which revealed the fact that there must be a central pneumonia. Acting upon this report, they discovered the signs of pneumonia, which were found to be present and fortunately for the patient the operation was not performed.

A woman in St. Louis City Hospital who was septic was ordered to be put on a streptococci vaccine. A blood culture being performed, and the live steptococcus found in the blood it was deemed more wise to give her antibodies already formed, that is, an antistreptococcic serum. Good results were obtained which could not have been accomplished by pouring into the circulation dead organisms, when the body was full of the live ones, showing that the antibodies were broken down to such an extent that she needed antibodies, that is, antistreptococcic serum instead of more of the same thing that had already overcome the fighting powers of the patient's serum.

In a large western city, two years ago, blood cultures were hardly ever asked for, but now a laboratory man there who specializes on this branch keeps a laboratory force busy working out the cultures which he is called on to make. The physicians there are seeing more and more the uses blood cultures may be put to.

If a city is in the grip of a typhoid epidemic nowadays, people don't stand and helplessly wring their hands and ask where it comes from. They study the sections where it is most prevalent, and knowing it is a food or water borne infection, the water is examined, and also the food, which is generally milk. The milk of the dealer supplying these people is examined with the differential media, which now makes the study of typhoid easy. The family of the dealer, if need be, are examined to see if there is a possible carrier among them. The methods of the washing of the bottles are examined. The washwater itself is cultured until the enemy is traced to its lair and the possibility for its continuing to infect the people is destroyed.

If a patient is suffering from the vomiting of pregnancy, the question as to whether that vomiting is caused by nerve reflexes or by toxaemia is determined by the presence of a small amount of amonia in the urine. If albumen is found in the urine of a patient or if not, and there is a high blood pressure, we don't sit down and say, "That person has nephritis. It is all up with him." We find out how much of his kidneys are destroyed, by the functional test and we act accordingly. Also the examination of the urine reveals to us by the presence of indican, etherial sulphates, urea, etc., how our treatment of toxic conditions is progressing.

An examination of the urine may show the presence or absence of disease in the pancreas, so by intelligent handling of these various fluids, the jealously guarded secrets of the hidden organs are revealed, as the X-ray reveals the leaden messenger of death sent on its mission by the assassin's hand. The finding of these lesions is just as important and needs the trained specialist just as much as we need the Rhoentgenologist's in the use of his wonderful rays, to find the bullet.

But then some men will go forever in their indifferent and ignorant ways. As a "Doc" said recently, "I don't have much use for them laboratories. I find the great majority of my patients git along best with a dose of calomel and salts and a good letting alone." I agreed with him, for I thought the more he tried to interfere with nature, the greater mess he would make of it.

How many cancers, so called, would never have been operated if a Wassermann had been done? How many deadly epidemics of diphtheria would have been prevented, had a culture been made of the first sore throats to appear in the commuity? The Diety, to quote the country on the subject, "has seen fit in His wisdom" to remove many a child from our midst in the past, who might have been saved by a dose of anti-toxin had an alert diagnostician been on the job.

Then who are the men who use the laboratories—the ignorant man? No! He knows not that he knows not, and is consequently blissfully happy in the conceit, that he is the wizard the undiscriminating public takes him to be His sun is setting, but he still may be found.

The partially educated man? No! Because he does not wish to acknowledge that he is only partially educated.

It is the alert man, the man who is constantly consulted because of his wisdom and skill. This man is in earnest, he is searching for the uttermost rays of light that can be thrown on his case. He knows the greatest light he can secure may be but little, so he knows he needs it, and when and where he needs such help. He is discriminating. He goesn't blindly give a stock of vaccine without knowing absolutely what organism is causing the trouble, as so many ignorantly do.

Such men are growing in numbers, thanks to good schools, and the Law demands that a man shall know some of the rudiments of medicine before he can practice. Consequently laboratories are increasing in numbers with the increasing demand for these men. The laboratories are even beginning to evidence signs of prosperity, so that in the distant future the laboratory man may too perhaps be seen in his brightly polished and expensive machine, which we are credibly informed the public demands of the physician.

---

A five per cent. solution of phenol in glycerine dropped warm into the ear will relieve the pain in non-suppurating acute otitis media.—*American Journal of Surgery.*

---

When removing a dermoid cyst at the root of the nose don't forget that it may lead through the bone sutures to the meninges. Especially if the cyst or sinus is infected it is not wise to dissect it out too deeply unless persistent discharge after removal of the presenting portion cannot be cured by cauterization, etc.—*American Journal of Surgery.*

